# Potentiation of apoptosis by histone deacetylase inhibitors and doxorubicin combination: cytoplasmic cathepsin B as a mediator of apoptosis in multiple myeloma

**DOI:** 10.1038/bjc.2011.42

**Published:** 2011-03-01

**Authors:** V Cheriyath, M A Kuhns, M E Kalaycio, E C Borden

**Affiliations:** 1 Translational Hematology and Oncology Research, Taussig Cancer Institute, Cleveland Clinic, Cleveland, OH 44195, USA; 2 Department of Hematology Oncology and Blood Disorders, Taussig Cancer Institute, Cleveland Clinic, Cleveland, OH 44195, USA

**Keywords:** HDAC inhibitors, cathepsin B, doxorubicin, apoptosis and multiple myeloma

## Abstract

**Background:**

Although inhibitors of histone deacetylase inhibitors (HDACis) in combination with genotoxins potentiate apoptosis, the role of proteases other than caspases in this process remained elusive. Therefore, we examined the potentiation of apoptosis and related mechanisms of HDACis and doxorubicin combination in a panel of myeloma cell lines and in 25 primary myelomas.

**Results:**

At IC_50_ concentrations, sodium butyrate (an HDACi) or doxorubicin alone caused little apoptosis. However, their combination potentiated apoptosis and synergistically reduced the viability of myeloma cells independent of p53 and caspase 3–7 activation. Potentiated apoptosis correlated with nuclear translocation of apoptosis-inducing factor, suggesting the induction of caspase 3- and 7-independent pathways. Consistent with this, butyrate and doxorubicin combination significantly increased the activity of cytoplasmic cathepsin B. Inhibition of cathepsin B either with a small-molecule inhibitor or downregulation with a siRNA reversed butyrate- and doxorubicin-potentiated apoptosis. Finally, *ex vivo,* clinically relevant concentrations of butyrate or SAHA (suberoylanilide hydroxamic acid, vorinostat, an HDACi in clinical testing) in combination with doxorubicin significantly (*P*<0.0001) reduced the survival of primary myeloma cells.

**Conclusions:**

Cathepsin B has a prominent function in mediating apoptosis potentiated by HDACi and doxorubicin combinations in myeloma. Our results support a molecular model of lysosomal–mitochondrial crosstalk in HDACi- and doxorubicin-potentiated apoptosis through the activation of cathepsin B.

Despite recent advances, disease recurrence and side effect profiles of existing drugs remain a problem in multiple myeloma (Ludwig *et al*, 2010; Niesvizky and Badros, 2010; Richards and Weber, 2010). As evasion of apoptosis may be important for early expansion and accumulation of mutant plasma cells, identification of drug combinations that potentiate apoptosis might be beneficial for achieving better outcomes (Bergsagel and Kuehl, 2001; Cheriyath *et al*, 2007b).

Besides caspases, proteolysis is mediated by lysosomal cathepsins such as cathepsin B and cathepsin D in apoptosis (Boya *et al*, 2003; Broker *et al*, 2004; Paquet *et al*, 2005; He *et al*, 2005a; Turk and Stoka, 2007). Once released from lysosomes, cathepsins may contribute to apoptosis execution either by direct cleavage of cellular substrates, by acting in concert with caspases or by disrupting the mitochondrial transmembrane potential (ΔΨ) (Boya *et al*, 2003; Broker *et al*, 2004; Paquet *et al*, 2005; He *et al*, 2005a; Turk and Stoka, 2007). Various insults including oxidative stress and DNA damage may lead to the limited release of cathepsins that culminate in the induction of apoptosis. However, the release of cathepsin in excess may lead to cellular necrosis (Paquet *et al*, 2005).

Cancer cells evade apoptosis by both genetic (mutations) and epigenetic means (Egger *et al*, 2004). Perturbation of the balance between histone acetyltransferases and histone deacetylases (HDACs) has been defined in myeloma and in other cancers (Mitsiades *et al*, 2003; Marks and Xu, 2009; Luszczek *et al*, 2010). Consequently, inhibition of HDACs has emerged as a potential therapeutic strategy (Glaser, 2007; Richon *et al*, 2009). Histone deacetylase inhibitors (HDACis) include several classes, ranging from the simple aliphatic acid sodium butyrate (butyrate) to more complex hydroxamic acid-derived compounds, such as SAHA (suberoylanilide hydroxamic acid, vorinostat; Moradei *et al*, 2008; Balliet *et al*, 2009). Histone deacetylase inhibitors cause cytotoxicity of cancer cells epigenetically by re-expressing silenced tumour suppressors (Laird, 2005; Glaser, 2007; Richon *et al*, 2009) or by altering acetylation status of cellular proteins through non-epigenomic mechanisms (Pei *et al*, 2004; Dai *et al*, 2005; Chen *et al*, 2007).

Toxicities associated with cumulative or higher dosages limit HDACi usage as a monotherapy (Richardson *et al*, 2008; Badros *et al*, 2009; Richon *et al*, 2009). To overcome this limitation, synergistic interaction of HDACi with a variety of anticancer agents including anthracyclins, which inhibits topoisomerases I and II, have been explored (Marchion *et al*, 2004; Mitsiades *et al*, 2004; Catalano *et al*, 2006; Pan *et al*, 2007; Hajji *et al*, 2008; Sanchez *et al*, 2010). In myeloma cell lines, at above IC_50_ concentrations, HDACi (SAHA and LBH589) and anthracyclins potentiate apoptosis through a variety of mechanisms, including the induction of caspase 3 and 7 or by altering the expression of proapoptotic genes (Mitsiades *et al*, 2004; Sanchez *et al*, 2010). However, the molecular mechanisms of the synergy between HDACi and doxorubicin at their IC_50_ or sub-IC_50_ concentrations are not known. In this study, we identified a critical role for cathepsin B in mediating the potentiation of apoptosis in myeloma cells by HDACi and doxorubicin combinations at their IC_50_ concentrations. Moreover, both experimental (butyrate) and a clinically relevant HDACi (vorinostat) and liposomal doxorubicin (doxil) significantly reduced the viability of patient-derived primary myeloma cells, providing a rationale of combining this non-neurotoxic and steroid sparing combination for myeloma and other haematological malignancies.

## Materials and methods

### Cell lines

Human multiple myeloma cell lines RPMI 8226, U266 and NCI-H929 were purchased from the ATCC (Manassas, VA, USA); KMS-11 and OPM-2 cells were kind gifts from Drs Taolin Yi and Eric Hsi of the Cleveland Clinic. All cells were propagated in recommended media.

### Reagents and antibodies

Sodium butyrate was purchased from Sigma (Sigma-Aldrich, St Louis, MO, USA), SAHA (vorinostat) was provided by Merck & Co. Inc., (Whitehouse Station, NJ, USA) and liposomal doxorubicin (Doxil) was obtained from the Cleveland Clinic Cancer Pharmacy. Cathepsin B inhibitor CA-074Me was obtained from Calbiochem (Calbiochem Inc., San Diego, CA, USA) and caspase 3 inhibitor DEVD-CHO and pan-caspase inhibitor z-VAD-FMK were from BioMol Inc., (Plymouth Meeting, PA, USA). Antibodies for caspase 3 and apoptosis-inducing factor (AIF) were from Cell Signaling Technology Inc., (Danvers, MA, USA). Cathepsin B antibody was from Sigma (Sigma-Aldrich). Cathepsin stealth siRNA was from Invitrogen Inc (Carlsbad, CA, USA).

### Viability assays and synergy analysis

Human multiple myeloma cell lines were seeded in 96-well plates at a concentration of 5 × 10^3^ cells per well and incubated for 72 h with various concentrations of butyrate, doxorubicin or their combinations. At the end of incubation, the percentage reduction in cell viability compared with untreated cells was determined with Alamar blue (Invitrogen Inc.) as described (Cheriyath *et al*, 2007a). The median drug effect for butyrate, doxorubicin and their combinations was analysed using Calcusyn software (Chou and Talalay analysis) to derive the combination indices.

Informed consent for bone marrow (BM) aspirates was obtained in accordance with protocols approved by the Institutional Review Board of Cleveland Clinic (Cleveland, OH, USA). The effect of HDACi, doxorubicin or their combinations on the survival of fresh myeloma cells after drug treatments was measured as previously described (Lincz *et al*, 2001; Cheriyath *et al*, 2007a).

### Apoptosis and caspase 3 and 7 activity assay

TUNEL labelling (BD Biosciences, San Jose, CA, USA) and caspase 3 and 7 activities (Caspase-Glo assay reagent, Promega Inc., Madison, WI, USA) were measured according to manufacturer's instructions and as described (Cheriyath *et al*, 2007a; Bae *et al*, 2008).

### Cathepsin B, cathepsin D and calpain assays

Cathepsin B, cathepsin D and calpain activity kits were used to measure enzymatic activity according to manufacturer's instructions (Biovision Inc., Mountain View, CA, USA). Briefly, 1 × 10^6^ cells were treated with butyrate, doxorubicin or their combination for the indicated time periods. Cytoplasmic cathepsin B activity was assessed in cytoplasmic extracts prepared by permeabilising the plasma membrane with digitonin containing buffer (50 *μ*g ml^−1^ digitonin, 250 mM Sucrose, 20 mM Hepes, 10 mM KCl, 1.5 mM MgCl2, 1 mM EDTA, 1 mM EGTA, 1 mM Pefablock, pH 7.5) for 10 min on ice. A measure of 15 *μ*l of cytoplasmic extract was then diluted with 35 *μ*l of assay buffer and incubated with AFP-conjugated substrates for 1 h. The increase in fluorescence was measured with a Wallac Victor2 fluorimeter (Waltham, MA, USA). Lactate dehydrogenase (LDH) activity using cytotox 96 well assay kit (Promega Inc.) was used to monitor permeabilisation of plasma membrane and to normalise the activity of cytoplasmic cathepsin B.

### Indirect immunofluorescence microscopy

Cells left untreated or treated with butyrate, doxorubicin or their combination for the indicated time periods were cytospun and fixed with 4% paraformaldehyde for 10 min. Fixed cells were permeabilised with 0.2% Triton X-100 for 10 min and incubated with monoclonal anti-AIF antibody (dilution of 1 : 200) for 1 h followed by secondary antibody conjugated with Alexa-488 (dilution 1 : 1000; Invitrogen Inc.). Cells were then mounted with Prolong Gold mounting media with DAPI and imaged using a Leica DMI4000B fluorescence microscope (Leica, Bannockburn, IL, USA).

### Immunoblot analysis

Whole-cell extracts (WCE) were made by lysing 1 × 10^6^ cells with RIPA buffer (Sigma-Aldrich) containing 1 × protease inhibitor cocktail (Calbiochem). A quantity of 25–35 *μ*g of WCE was subjected to immunoblot analysis as previously described (Cheriyath *et al*, 2007a; Bae *et al*, 2008).

### siRNA-mediated downregulation of cathepsin B

Cells (1 × 10^6^) were transfected with cathepsin B stealth siRNA by Lipofectin according to manufacturer's instructions (Invitrogen Inc.). After 24 h transfection, cells were treated with butyrate, doxorubicin or their combination for 72 h.

### Statistical analysis

One-way repeated measures ANOVA followed by all pairwise multiple comparison procedures (Holmes-Sidak method) were performed using SigmaStat 3.5 software to determine the significance of difference between untreated, single agent and combination-treated fresh myeloma samples. One-way ANOVA followed by Tukey's Multiple Comparison Test was used to determine the significance of difference of cathepsin B activity and potentiation of apoptosis between the untreated and treated samples. Two-tailed *t*-test was used to determine the significance of difference of the effects of caspase 3 and cathepsin B inhibitors on apoptosis.

## Results

### HDACi and doxorubicin combination potentiated apoptosis in myeloma cells

**Figure 1:**
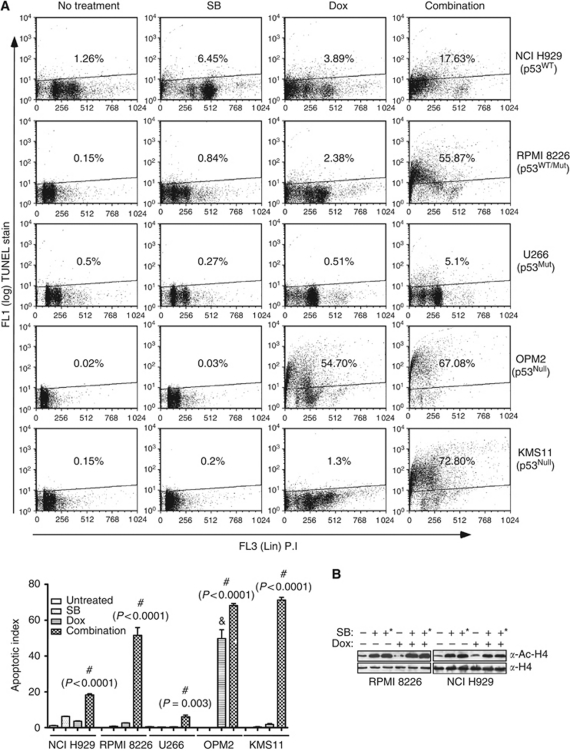
Combinations of HDACi and doxorubicin potentiate apoptosis in myeloma cells. (**A**) Effects of HDACi, doxorubicin and their combination on apoptosis of myeloma cells with varying p53 status. Myeloma cells (1 × 10^6^) carrying either wild-type or mutant p53 (NCI H929, RPMI 8226, U266, KMS11 and OPM2) were left untreated or treated with butyrate (300 *μ*M for NCI H929 and 600 *μ*M for RPMI 8226, U266, KMS 11 and OPM2) or doxorubicin (40 nM) or with their combination for 48 h. Percentage of cells undergoing apoptosis was assessed by TUNEL staining. Scatter plot shown is a representative of two independent experiments with similar results, in which 10 000 events were collected using flow cytometry. Mutational status of p53 of the myeloma cell lines is indicated. Induction of apoptosis in myeloma cells by butyrate, doxorubicin and their combination are summarised in the bottom graph. Each bar on the graph is mean±s.e.m. of two independent experiments. # Indicates that the treatment is significantly different from other treatments and ‘&’ sign indicates that treatment is significantly different from untreated or butyrate treatment; *P*-values for each treatment is provided. (**B**) Effect of butyrate and doxorubicin (40 nM) combination on HDAC activity. Whole-cell lysates (WHL; 30 *μ*g) of NCI-H929 or RPMI 8226 cells left untreated or treated with butyrate (+=300 *μ*M, +^*^=600 *μ*M) doxorubicin or their combination for 36 h and acetylation status of histone H4 as an indirect measure of HDAC activity was determined by immunoblot analysis.

To assess whether the combination of butyrate and doxorubicin potentiate apoptosis at their IC_50_ or sub-IC_50_ concentrations, five myeloma cell lines carrying wild-type or mutant p53 alleles were treated with either butyrate, doxorubicin alone or with their combination for 48 h, and apoptotic indices were assessed by TUNEL assay (Figure 1A). In initial studies, the IC_50_ of butyrate, SAHA and doxorubicin were determined in NCI H929, RPMI 8226 and U266 cells (Supplementary Figure 1 and Supplementary Table 1). Butyrate or doxorubicin alone had only a modest effect on TUNEL positivity. However, their combination markedly increased TUNEL staining in all myeloma cell lines tested irrespective of p53 status (Figure 1A). An exception to this was OPM2 cells, in which doxorubicin alone resulted in 54.7% positive TUNEL staining (Figure 1A). These results suggested that potentiation of apoptosis by the combination may lead to synergistic reduction in the viability of myeloma cells.

As augmented inhibition of HDACs could lead to potentiation of apoptosis, effects of butyrate, doxorubicin and their combination on HDAC activity was tested by assessing histone-H4 acetylation. As expected, butyrate induced the acetylation of histone H4 in RPMI 8226 and NCI-H929, indicating the inhibition of HDACs (Figure 1B). However, acetylation status of histone H4 was unaltered by doxorubicin alone or in combination with butyrate (Figure 1B). These results suggest that potentiated apoptosis by the combination is not resulting in from the augmented inhibition of HDACs.

In agreement with the potentiated apoptosis, combinations of HDACi and doxorubicin synergistically reduced viability of myeloma cell lines (Supplementary Figure 2). Compared with single agents, butyrate (150, 300 and 600 *μ*M) and doxorubicin (15, 30 and 60 nM) co-treatment with incrementally increased concentrations markedly reduced the viability of myeloma cells. In all three cell lines, butyrate and doxorubicin combinations resulted in a combination index of <1 in Chou and Talalay analysis, indicating synergistic interaction between them (Supplementary Figure 2A). To understand the relative contribution of HDACi to the synergy, myeloma cells were treated with a fixed concentration of doxorubicin (40 nM) and varying concentrations of butyrate (0–800 *μ*M). Similar to incrementally increased concentrations of butyrate and doxorubicin, increased concentrations of butyrate with a constant concentration of doxorubicin also resulted in synergy with a combination index of <1 (Supplementary Figure 2B). As in co-treatment, sequential treatment (SAHA followed by doxorubicin) also synergistically reduced the viability of myeloma cell lines (data not shown).

### HDACi and doxorubicin significantly reduced viability of patient-derived fresh myeloma cells

To define the clinical relevance of HDACi and doxorubicin combination, the antimyeloma activity of the clinically relevant concentrations of HDACi and doxorubicin combinations was tested in patient-derived primary myeloma cells (*n*=25). Characteristics of the patients and treatments that they received before the collection of BM aspirates are provided (Supplementary Table 2). CD138^+^ cells derived from BM aspirates of 18 myeloma patients were left untreated or treated with butyrate, doxorubicin or their combination in the presence of BM mononuclear cells, which includes stromal cells. After 72 h of treatment, survival of CD138^+^ cells was assessed by flow cytometry. Compared with untreated cells, butyrate (600 *μ*M) or doxorubicin (40 nM) alone had only a marginal effect on the survival of CD138^+^ cells (100% untreated *vs* 72.97% butyrate and 83.25% doxorubicin treated; Table 1). However, co-treatment of butyrate and doxorubicin significantly reduced the survival of CD138^+^ cells (43.42%, *P*⩽0.05; Table 1). While butyrate (600 *μ*M) and doxorubicin (40 nM) reached IC_50_ in 4 out of 18 and 1 out of 18 patient samples, respectively, their combination reached IC_50_ in 10 out of 18 patient samples.

Effect of combinations of HDACi and doxorubicin on the survival of fresh myeloma cells was further investigated using SAHA in BM aspirates from 12 myeloma patients. In initial studies, the IC_50_ of SAHA for myeloma cell lines ranged from 546 to 976 nM (Supplementary Table 1). Compared with single agents, combination of SAHA (200 nM) and doxorubicin (40 nM) markedly reduced viability of fresh myeloma cells. At the concentrations used, neither SAHA nor doxorubicin reached IC_50_ or IC_25_, but, combining SAHA with doxorubicin reached IC_50_ in 5 out of the 12 samples and IC_25_ in 11 out of the 12 samples (Table 1). In one-way repeated measures of ANOVA, the sub-IC_50_ concentration of SAHA used had only a marginal effect on the survival of fresh myeloma cells (CD138^+^; 80.87% in SAHA treated *vs* 100% in untreated). However, combining SAHA with doxorubicin significantly reduced the survival of fresh myeloma cells to 46.29% (*P*⩽0.05; Table 1).

### HDACi- and doxorubicin-potentiated apoptosis was caspase 3 and 7 independent

**Figure 2:**
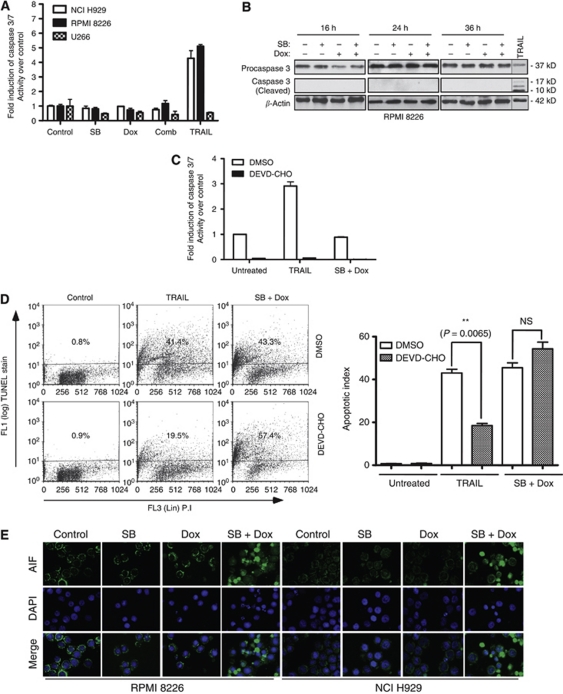
Effects of butyrate, doxorubicin or their combination on caspase 3 and 7 activation and AIF release in myeloma cells. (**A**, **B**) Effects of butyrate, doxorubicin and their combination on caspase 3 and 7 activation. (**A**) NCI H929, RPMI 8226 and U266 cells were treated with butyrate (SB; 300 *μ*M for NCI H929 and 600 *μ*M for RPMI 8226 and U266), doxorubicin (Dox; 40 nM) or with their combination. After 24 h treatments, fold change in caspase 3 and 7 activity relative to untreated cells was assessed by caspase 3 and 7 glo kit (Promega Inc.). TRAIL (50 ng ml^−1^, Peprotech Inc., Rocky Hill, NJ, USA) was used as a positive control. Each data point in the bar graph is mean±s.e.m. of three independent experiments performed in triplicate. (**B**) Caspase 3 cleavage was assessed after 16, 24 or 36 h by subjecting 30 *μ*g of whole-cell lysates (WCL) of RPMI 8226 cells to immunoblot analysis with a caspase 3-specific antibody. TRAIL-treated sample was used as a positive control and *β*-Actin as a loading control. (**C**, **D**) Effects of caspase 3 inhibitor DEVD-CHO on butyrate- and doxorubicin-induced apoptosis of myeloma cells. RPMI 8226 cells (1 × 10^6^) were pretreated with either vehicle (DMSO) or 1 *μ*M of cell permeable caspase 3-specific inhibitor DEVD-CHO (Biomol Inc.) for 2 h. Then the cells were left untreated or treated with TRAIL (50 ng ml^−1^) or butyrate (600 *μ*M) plus doxorubicin (40 nM). Caspase 3 and 7 activity was determined as in Figure 3A, and percentage of cells undergoing apoptosis was determined 48 h post-treatment by TUNEL assay as in Figure 2. Scatter plot shown is one of two independent experiments with similar results, in which 10 000 events were collected (top panel). Each bar on the graph is mean±s.e.m. of two independent experiments, and *P*-values of significantly different treatments are provided. (**E**) Butyrate plus doxorubicin combination results in nuclear translocation of AIF in RPMI 8226 and NCI H929 cells. RPMI 8226 or NCI H929 cells were left untreated or treated with indicated concentrations of butyrate, doxorubicin or their combination for 48 h. The localisation of AIF was assessed by indirect immunofluorescence staining with an AIF antibody followed by Alexa Flour-488-conjugated secondary antibody (Green staining). Nuclei of the cells were stained with DAPI (blue). Merged images were produced by superimposing both images. Results shown are representative of three independent experiments with similar results.

To gain a better understanding of the mechanism of apoptosis potentiated by butyrate and doxorubicin, effects of these agents alone or in combination on caspase-dependent and -independent apoptosis were investigated. Butyrate and doxorubicin treatments had no marked effect on the activity of caspase 3 and 7 in NCI H929, RPMI 8226 and U266 cell lines at 24 h (Figure 2A). Under the same conditions TRAIL, a potent inducer of apoptosis, markedly increased the activity of caspase 3 and 7 in sensitive cell lines (NCI H929 and RPMI 8226), suggesting the absence of an intrinsic block in caspase activation pathways in these cell lines. Lack of caspase 3 activation was confirmed by caspase 3 cleavage assay at 16, 24 and 36 h (Figure 2B). The role of caspase 3 in apoptosis potentiated by butyrate and doxorubicin was further tested by pretreating RPMI 8226 cells with DEVD-CHO, a cell permeable caspase 3-specific peptide inhibitor. DEVD-CHO inhibited the activity of caspase 3 and 7 (Figure 2C). Consistent with its inhibition of caspase activity, DEVD-CHO significantly reduced the apoptosis induced by TRAIL from 41.4 to 19.5% (*P*=0.0065); however, it had no apparent effect on apoptosis potentiated by the butyrate and doxorubicin combination (Figures 2C and D). Influence of caspases other than caspase 3 and 7 on butyrate and doxorubicin combination potentiated apoptosis was tested using z-VAD-FMK, a pan-caspase inhibitor. Compared with vehicle-treated cells, ∼12% decrease in apoptosis was observed in z-VAD-FMK-treated cells (Supplementary Figure 3). Together, these results suggest the involvement of caspases other than 3 and 7 in the potentiation of apoptosis by HDACi and doxorubicin.

As apoptosis potentiated by butyrate and doxorubicin was not caspase 3 and 7 dependent, nuclear translocation of AIF, a mediator of caspase-independent apoptosis, was assessed in RPMI 8226 and NCI H929 cells using indirect immunofluorescence microscopy. In untreated, butyrate- or doxorubicin-treated RPMI 8226 and NCI H929 cells, most of the AIF was localised in mitochondria resulting in very little colocalisation of AIF with nuclear stain DAPI (Figure 2E). However, the combination of butyrate and doxorubicin markedly increased the nuclear translocation of AIF in both RPMI 8226 and NCI H929 cells (Figure 2E). These results together with the lack of caspase 3 and 7 activation suggested the involvement of caspase 3- and 7-independent pathways in mediating the apoptosis potentiated by HDACi and doxorubicin combinations.

### HDACi and doxorubicin significantly increased the activity of cytoplasmic cathepsin B

**Figure 3:**
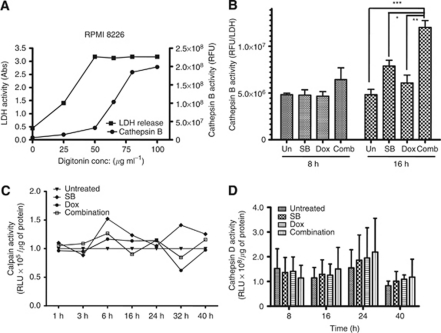
Combinations of butyrate and doxorubicin significantly increased the activity of cytoplasmic cathepsin B. (**A**) Optimisation of cytoplasmic cathepsin B extraction by permeabilisation of plasma membrane with digitonin. RPMI 8226 cells plasma membranes were permeabilised with increasing concentrations of digitonin for 10 min in ice. Permeabilisation of plasma membranes was monitored by assessing LDH activity (left *Y* axis) and permeabilisation of lysosomes was monitored by cathepsin B activity (right *Y* axis). Each point on the graph is mean±s.e.m. of two independent experiments. (**B**) Effects of butyrate and doxorubicin combination on the activity of cytoplasmic cathepsin B in myeloma cells. RPMI 8226 cells were treated with butyrate (SB, 600 *μ*M), doxorubicin (Dox, 40 nM) or their combination. Cells were harvested at indicated time periods, permeabilised with 50 *μ*g ml^−1^ digitonin, and the activity of cathepsin B was measured using enzyme assay kits (Biovision Inc.). Cathepsin B activity was normalised to LDH activity; ^*^P<0.05, ^**^P<0.001 and ^***^P<0.0001. (**C**, **D**) Effects of butyrate and doxorubicin combination on the activity of calpain and cathepsin D in RPMI 8226 cells. Relative increase in total calpain (**C**) and cathepsin D (**D**) activities were calculated by normalising to untreated samples. Each data point on the graph is mean±s.e.m. of two independent experiments performed in triplicate.

On the basis of the role of cathepsin B in mediating AIF activation and apoptosis induced by agents that act on DNA (Bidere *et al*, 2003; Broker *et al*, 2004; Biswas *et al*, 2005), we postulated that increased activity of cathepsin B in the cytoplasm may be a critical mediator of HDACi and doxorubicin combination induced apoptosis. To test this hypothesis, activity of cathepsin B in cytoplasmic extracts of untreated and treated RPMI 8226 cells was determined. Permeabilisation of cells with digitonin was monitored by measuring the activity of cytoplasmic enzyme LDH (Foghsgaard *et al*, 2002). Incubation of cells in cytoplasmic extraction buffer with 50 *μ*g of digitonin resulted in the maximal release of LDH with minimal increase in the activity of cathepsin B, indicating the permeabilisation of the plasma membrane, but not lysosomes (Figure 3A). In kinetic studies compared with untreated and single agents, the combination of butyrate and doxorubicin significantly increased the cytoplasmic activity of cathepsin B at 16 h in RPMI 8226 cells (Figure 3B). An increase in the activity of cytoplasmic cathepsin B was also observed in NCI H929 cells treated with butyrate and doxorubicin combination (data not shown).

As other lysosomal enzymes, including cathepsin D and calpain, are also suggested to be involved in HDACi and stress-induced apoptosis, combination-mediated activation of cathepsin D and calpains was assessed in RPMI 8226 cells (Mandic *et al*, 2002; Carew *et al*, 2007). Unlike cathepsin B, butyrate and doxorubicin combination failed in activating cathepsin D and calpain (Figures 3C and D).

### Cathepsin B inhibition reverses HDACi and doxorubicin combination potentiated apoptosis

**Figure 4:**
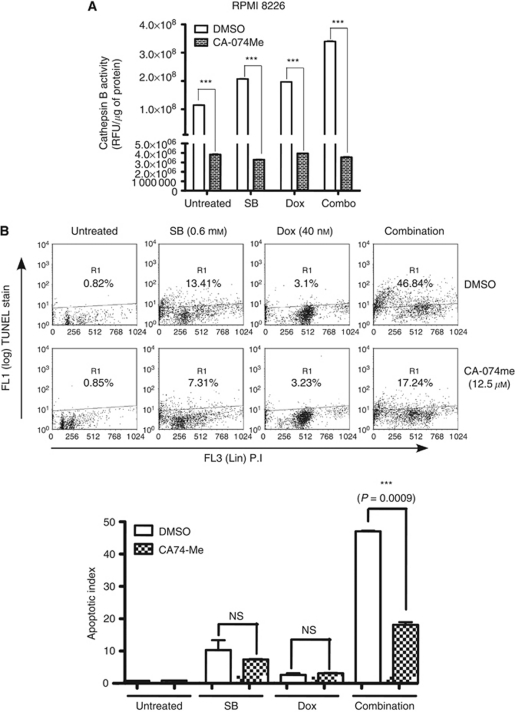

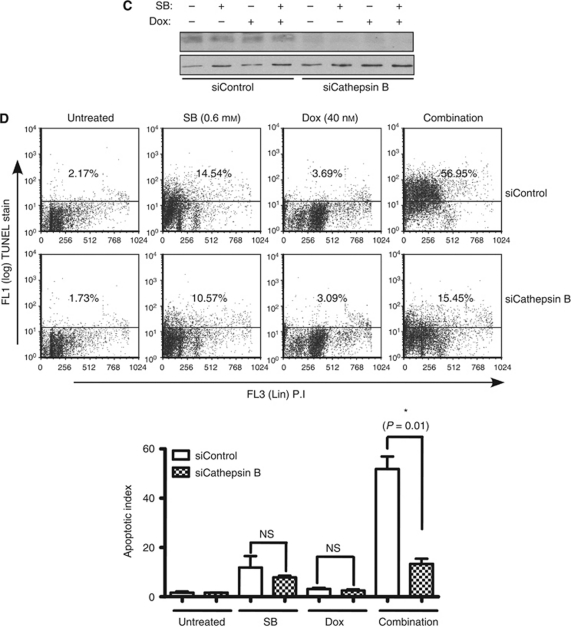
Cathepsin B inhibitor attenuated butyrate- and doxorubicin-induced apoptosis in RPMI 8226 cells. (**A**) Cathepsin B inhibitor attenuated butyrate- and doxorubicin-mediated activation of cathepsin B in RPMI 8226 cells. RPMI 8226 cells were treated with cell permeable cathepsin B-specific inhibitor CA-074Me (12.5 *μ*M) or vehicle (DMSO) for 1 h before treatment with butyrate (SB; 0.6 mM), doxorubicin (Dox; 40 nM) or combination. After 40 h treatment, cathepsin B activity was assessed using cathepsin B activity assay kit (Biovision Inc). Each data point on the graph is an average of two independent experiments performed in triplicate. (**B**) Cathepsin B inhibitor antagonised butyrate- and doxorubicin-induced apoptosis. After 72 h butyrate (SB; 0.6 mM), doxorubicin (Dox; 40 nM) or combination treatment, apoptosis indices in vehicle (DMSO)- or CA-074me (12.5 *μ*M)-pretreated cells were compared with TUNEL assay. Scatter plot shown is one of two independent experiments with similar results, in which 10 000 events were collected (top panel). Each bar on the graph is mean±s.e.m. of two independent experiments, and *P*-value of significantly different treatments is provided (bottom panel). (**C**, **D**) Downregulation of cathepsin B with a siRNA rescue RPMI 8226 cells from combination potentiated apoptosis. RPMI 8226 cells were transfected with either a scrambled siRNA (siControl) or a siRNA specific to cathepsin B (siCathepsin B). Knockdown of cathepsin B was determined by immunoblot analysis of 30 *μ*g of WHL 48 h post-transfection (**C**) and apoptotic index 72 h post-transfection by TUNEL assay (**D**). Each bar on the graph is mean±s.e.m. of two independent experiments.

As butyrate and doxorubicin combination markedly increased the activity of cytoplasmic cathepsin B, its role in potentiating apoptosis was further investigated in RPMI 8226 cells with CA-074me, a membrane-permeable cathepsin B inhibitor (Ostenfeld *et al*, 2005; Sandes *et al*, 2007; Wang *et al*, 2008). As expected, pretreatment of RPMI 8226 cells with CA-074me markedly inhibited total cathepsin B activity in control, butyrate, doxorubicin and combination treated cells (Figure 4A). Additionally, inhibition of cathepsin B activity with CA-074me significantly reduced the TUNEL positivity of RPMI 8226 cells treated with the combination from 46.84 to 17.24% (*P*=0.0009), suggesting a prominent role for cathepsin B in the mediation of butyrate- and doxorubicin-potentiated apoptosis (Figure 4B).

To test the direct role of cathepsin B in mediating the HDACi- and doxorubicin-potentiated apoptosis, its expression was downregulated in RPMI 8226 cells with a siRNA (Figure 4C). Compared with control siRNA-transfected cells, downregulation of cathepsin B reduced the butyrate and doxorubicin combination induced from 36.73 to 11.31% (Figure 4D). These results are in agreement with the results of using cathepsin B inhibitor CA-074me and confirm the role of cathepsin B in mediating the potentiation of apoptosis by butyrate and doxorubicin.

## Discussion

To further the current successes in myeloma therapy, there is a need to identify drug combinations that synergistically reduce viability of myeloma cells (Mitsiades *et al*, 2007; Richardson *et al*, 2007; Cheriyath *et al*, 2007b). Although anthracyclins (doxorubicin) have been used clinically as single agents or in combination to therapeutic advantage, greater effectiveness could improve activity. Moreover, the success of combination therapies could be improved by defining molecular mechanisms responsible for their antitumour activity, information that might be useful for patient selection and for predicting treatment outcomes.

Histone deacetylase inhibitors have been identified as epigenetic modulators and are in clinical trials either as single agents or in combination (Marks and Xu, 2009; Richon *et al*, 2009). We have identified potentiation of apoptosis and marked reduction of the viability of both myeloma cell lines and fresh myeloma cells by combinations of HDACi (butyrate and SAHA) and doxorubicin (Figure 1, Table 1 and Supplementary Figure 2), which is in agreement with other studies (Marchion *et al*, 2004; Mitsiades *et al*, 2004; Catalano *et al*, 2006; Sanchez-Gonzalez *et al*, 2006; Sanchez *et al*, 2010). However, this study identified a prominent role for cathepsin B in mediating HDACi- and doxorubicin-potentiated apoptosis at their IC_50_ or sub-IC_50_ concentrations. Interestingly, butyrate and doxorubicin potentiated apoptosis of myeloma cell lines, irrespective of p53 mutational status, and reduced the viability of fresh myeloma cells from patients who had been relapsed on a variety of therapies including liposomal doxorubicin (patient samples P02, P08, P15 and P16, Supplementary Table 1). These results suggested that HDACi and doxorubicin combinations could be useful for treating patients who recur on existing therapies and provide a rationale of testing this combination in clinics. In agreement with these results, *in vitro* and *in vivo* studies in mice have identified a synergistic antimyeloma effect for combinations of SAHA and the alkylating agents melphalan and doxorubicin (Campbell *et al*, 2010; Sanchez *et al*, 2010).

Lack of caspase 3 and 7 activation and the increased levels of nuclear AIF by butyrate and doxorubicin combination suggested the involvement of caspase-independent pathways in the potentiation of apoptosis in myeloma cells (Figure 2). Further investigation highlighted the importance of lysosomal cathepsin B in mediating apoptosis (Figures 3 and 4; Ivanova *et al*, 2008). Consistent with this, a cell permeable small-molecule inhibitor of cathepsin B or its downregulation with a siRNA rescued RPMI 8226 cells from potentiated apoptosis, suggesting a role for lysosomal cathepsin B in combination potentiated apoptosis (Figure 4).

A substantial reduction in apoptosis by a pan-caspase, but not caspase 3- and 7-specific inhibitor, suggests that potentiated apoptosis by HDACi and doxorubicin is a result of the concerted action of cathepsin B and caspases other than caspase 3 and 7 (Figure 2D and Supplementary Figure 3). Stresses acting on lysosomes could induce apoptosis by increasing the activity of cytoplasmic cathepsin B by various mechanisms, including (a) releasing the sequestered enzyme from lysosomes; (b) downregulating its negative regulators such as cystatin A; or (c) increasing the expression of its co-activators (Ivanova *et al*, 2008). It is unclear which of the above processes led to the combination mediated increased activity of cathepsin B. Once activated, cathepsin B could induce the cleavage of Bid, a proapoptotic member of Bcl2 family (Bidere *et al*, 2003; Biswas *et al*, 2005; Droga-Mazovec *et al*, 2008). Activated Bid could depolarise mitochondria releasing either cytochrome *c*, resulting in caspase-dependent apoptosis, or AIF and Endo G, leading to caspase-independent apoptosis (Boya *et al*, 2003). Increased cathepsin B activity in the cytoplasm also could lead to Bid-dependent or -independent activation of Bax by degrading its adaptor proteins, such as Ku70, Clusterin, Humanin and VDAC, that keep Bax in its inactive conformation (Guo *et al*, 2003; Zhang *et al*, 2005; Mazumder *et al*, 2007; Shoshan-Barmatz *et al*, 2008).

In summary, HDACi- and doxorubicin-potentiated apoptosis of myeloma cell lines was partly resulted in from activation of cathepsin B. Combination potentiated apoptosis and cathepsin B activity correlated with nuclear translocation of AIF, a mitochondrial sequestered proapoptotic factor involved in caspase-independent cleavage of DNA (Bidere *et al*, 2003). Enhanced accessibility of DNA to doxorubicin through chromatin relaxation, prolonged nuclear retention of doxorubicin and induction of tumour suppressors have been implicated in HDACi-mediated potentiation of apoptosis by anthracyclins (Catalano *et al*, 2006; Pan *et al*, 2007; Hajji *et al*, 2008). However, results of the current investigation support an apoptosis model involving lysosomal–mitochondrial crosstalk induced by the combinations of an epigenetic modulator and a DNA-damaging agent doxorubicin.

## Figures and Tables

**Table 1:** Effects of HDACi (butyrate or SAHA) and doxorubicin or their combinations on the survival of fresh myeloma cells

	**% Survival of CD138^+^ cells (treatments)**							
**Sample no.**	**Untreated**	**SB**	**Dox**	**Combo**	**Untreated**	**SAHA**	**Dox**	**Combo**
P01	100	65.6	92.6	51.1	—	—	—	—
P02	100	71.6	78.2	1.0	—	—	—	—
P03	100	70.1	76.9	37.8	—	—	—	—
P04	100	107.7	113.7	62.5	—	—	—	—
P05	100	33.9	85.3	18.9	—	—	—	—
P06	100	61.9	81.7	62.0	—	—	—	—
P07	100	104.3	93.3	85.7	—	—	—	—
P08	100	80.7	95.2	76.7	—	—	—	—
P09	100	102.2	101.5	92.7	100	85.7	97.8	88.1
P10	100	91.5	78.5	29.4	100	91.0	79.5	31.7
P11	100	59.5	65.5	52.5	—	—	—	—
P12	100	94.1	74.5	27.5	—	—	—	—
P13	100	39.1	85.8	19.4	—	—	—	—
P14	100	85.9	18.9	7.8	100	102.5	19.9	16.0
P15	100	43.9	74.2	20.5	—	—	—	—
P16	100	107.3	112.5	65.4	100	97.3	110.5	60.4
P17	100	60.6	73.3	47.7	—	—	—	—
P18	100	32.9	96.7	23.0	100	79.8	99.5	67.7
P19	—	—	—	—	100	83.2	87.8	73.1
P20	—	—	—	—	100	54.4	64.7	3.9
P21	—	—	—	—	100	88.3	101.3	56.2
P22	—	—	—	—	100	69.8	107.1	55.3
P23	—	—	—	—	100	41.3	87.5	16.5
P24	—	—	—	—	100	86.8	122.1	30.3
P25	—	—	—	—	100	81.4	92.9	51.4
Mean	100	72.9^#^	83.25^#^	43.42^***^	100	80.87^#^	89.73	46.29^***^
s.e.m.	0.00	5.96	4.93	6.35	0.00	5.40	7.78	7.57

Abbreviations: Dox=doxorubicin; HDACi=histone deacetylase inhibitor; SAHA=suberoylanilide hydroxamic acid; SB=sodium butyrate.

Mononuclear (1 × 10^5^) cells from bone marrow aspirates of patients were left untreated or treated with butyrate (600 *μ*M), SAHA (200 nM), doxorubicin (40 nM), butyrate plus doxorubicin or SAHA plus doxorubicin for 24 h. Percentages of dead and CD138+ positive cells were determined by flow cytometry, in which 10 000 events were collected for each treatment. Statistical significance between each treatment was determined by one-way repeated analysis of variance adjusted for multiple comparisons (Holm-Sidak method). ^#^ Significantly different from untreated and combination treated, *P*<0.0001; ^***^significantly different from untreated and single-agent-treated samples, *P*<0.0001.
