# Supplementary material for: Potentiation of apoptosis by histone deacetylase inhibitors and doxorubicin combination: cytoplasmic cathepsin B as a mediator of apoptosis in multiple myeloma
Source: Br J Cancer. 2011 Mar 1;104(6):957–67. doi: 10.1038/bjc.2011.42 (PMC3065279; doi:10.1038/bjc.2011.42)
Supplement: Supplementary Figures and Tables [file bjc201142x1.pdf]

Supplemental Figure 1

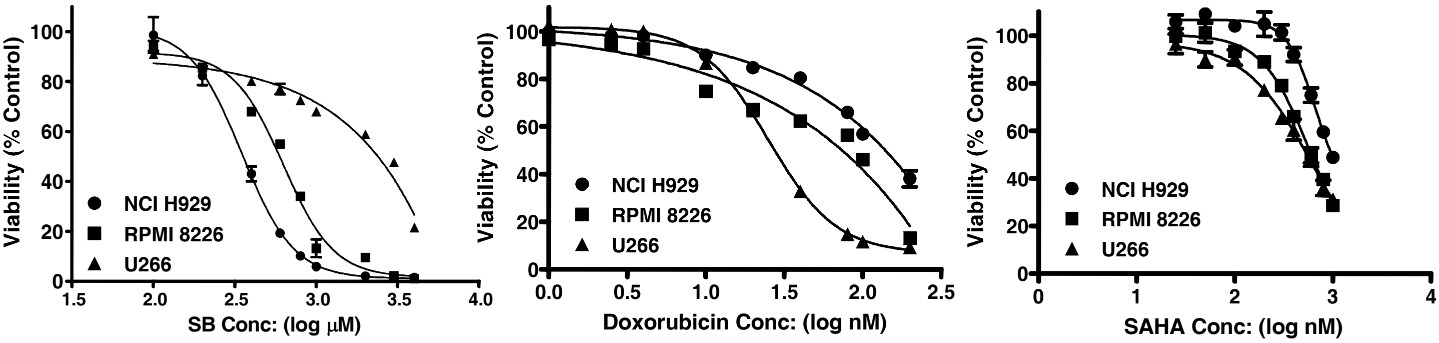

Supplemental Figure 2

A

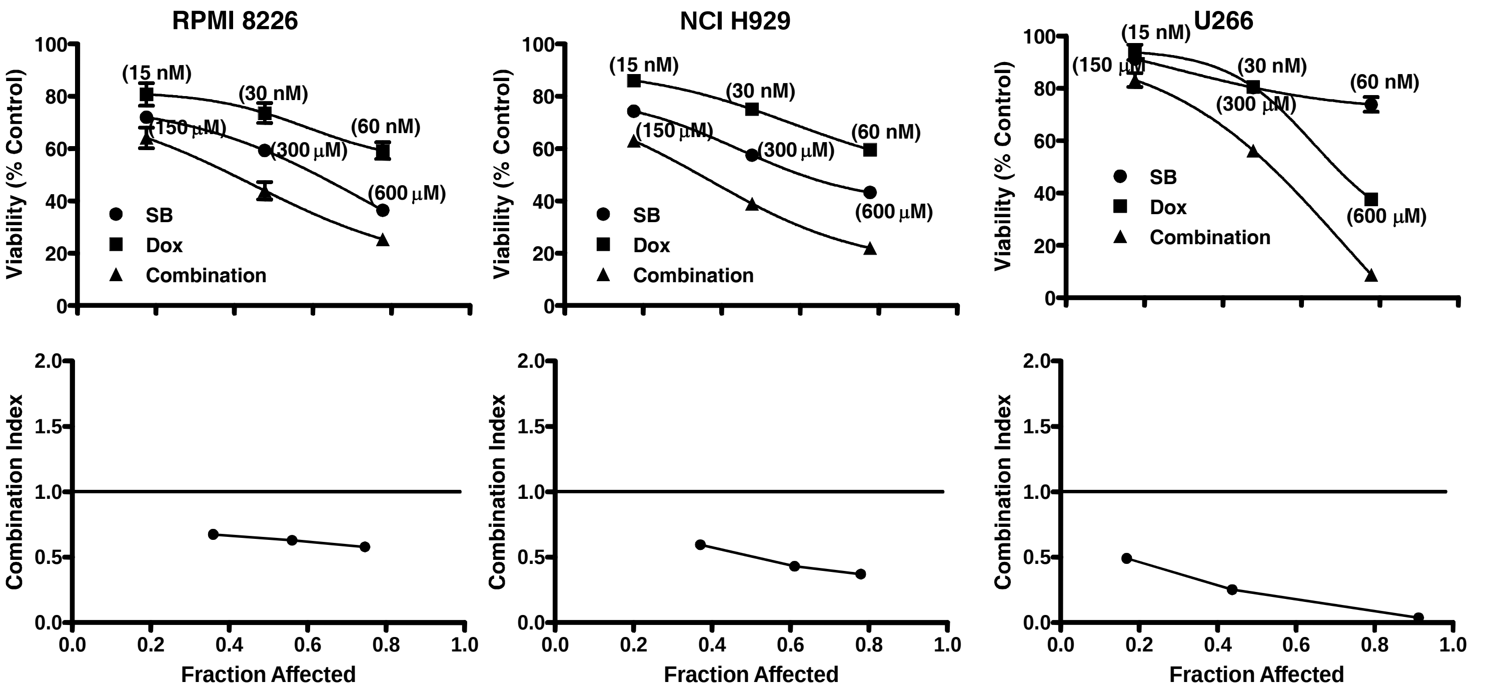

B

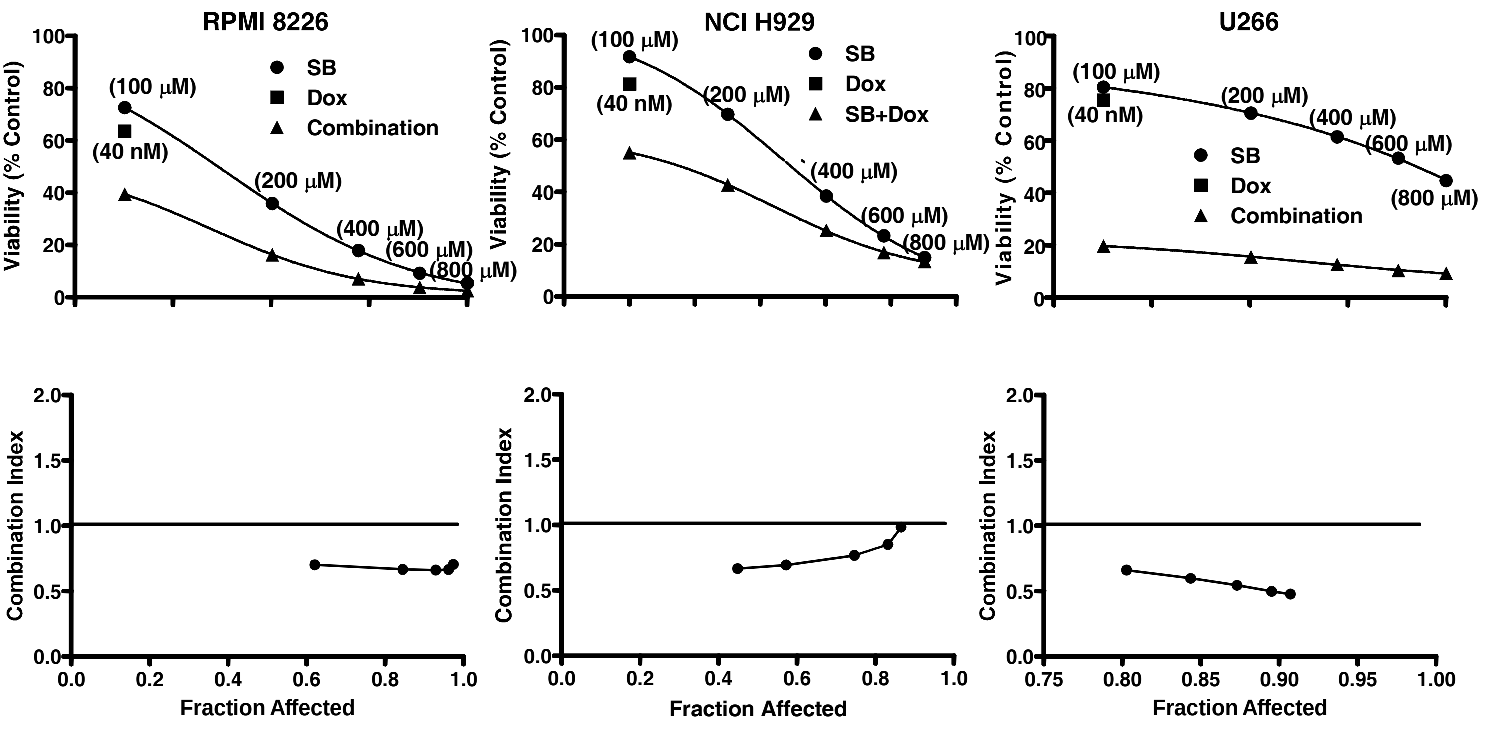

Supplemental Figure 3

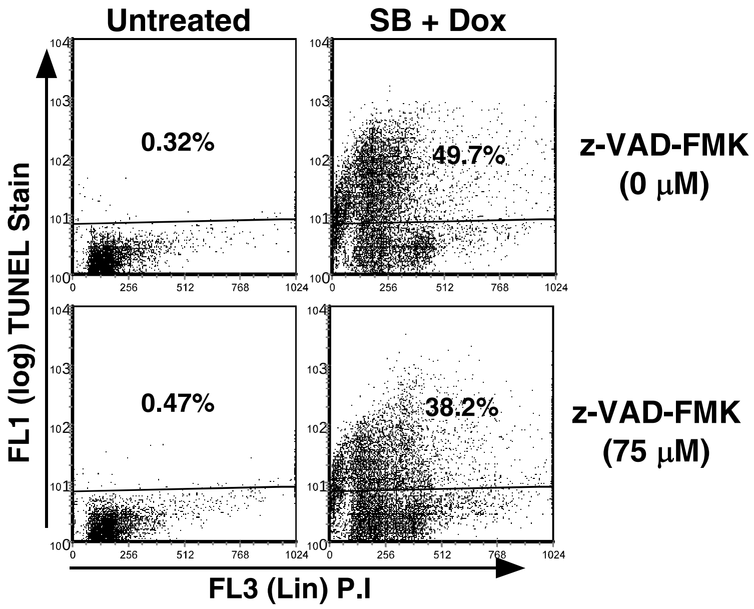

Supplemental Table 1

Cell Lines	IC_{50}		
	**SB (mM)**	**Doxorubicin (nM)**	**SAHA (nM)**
NCI H929	0.35	167	546
RPMI 8226	0.63	101	611
U266	0.90	29	976

Supplemental Table 2: Characteristics of myeloma patients

Sample #	Gender	Age	Disease Stage	Cytogenetics	Prior or Current Therapies
P01	Female	57	MM	ND	Cis, Eto, Ar
P02	Male	53	MM	ND	Doxil, Dex, Cyto, Rev
P03	Female	63	MM	ND	Rev, Dex
P04	Male	57	MM	ND	Dex
P05	Male	58	MM	ND	Thal, Pred, Rev, Dex
P06	Male	44	MM	ND	None
P07	Female	69	MM	ND	DVD-T, Ar, Pred
P08	Female	68	MM	ND	Vel, Rev, SAHA+Vel
P09	Female	54	PCL	Monosomy 13	Vel
P10	Female	42	MM, PCM	T(11;14)(q13;q32)	Cyto, Cis, Dec, Vel
P11	Male	56	MM	Normal	Reglan, Dec
P12	Male	78	MM	Normal	None
P13	Female	60	MM	t(4-14)	None
P14	Male	60	MM	Normal	Vel
P15	Female	51	MGUS	Normal	Cyto, Vel, Doxil, Rev
P16	Female	68	MM	17(p13)	Pred, Doxil, Vel
P17	Male	64	MM	Loss of Y	Vel
P18	Female	71	MM	Normal	None
P19	Male	71	MGUS	IgH(14q32)	Vel
P20	Male	61	MM, PCM	ND	Vel, Dex
P21	Male	61	MM	t(4-14)	Vel, Cyt, Thal
P22	Male	78	MM	Normal	Vel, Thal
P23	Female	60	MM	Normal	None
P24	Female	60	MM	ND	Rev
P25	Male	65	MM	ND	Thal

Abbreviations:
Disease stage: PCL - Plasma Cell leukemia, MM - Multiple Myeloma, PCM - Plasma cytoma, MGUS- Monoclonal gammopathy of undetermined significance.
Drugs: Vel-Velcade, Cis -Cisplatin, Dec-Decadron, Thal - Thalidomide, Rev - Revlimide, Vin - Vincristine, Pred-Prednisone, Doxil-liposomal doxorubicin.
