# Supplementary material for: Potentiation of apoptosis by histone deacetylase inhibitors and doxorubicin combination: cytoplasmic cathepsin B as a mediator of apoptosis in multiple myeloma
Source: Br J Cancer. 2011 Mar 1;104(6):957–67. doi: 10.1038/bjc.2011.42 (PMC3065279; doi:10.1038/bjc.2011.42)
Supplement: Supplementary Information [file bjc201142x2.doc]

**Supplemental Figure 1 and Supplemental Table 1: IC_50_ determination of Sodium Butyrate, doxorubicin and SAHA in myeloma cell lines.** 5x10^3^ myeloma cells (NCI H929, KMS11, OPM-2, RPMI 8226 and U266) were treated with increasing concentrations of butyrate (SB), SAHA or doxorubicin for 72 hrs and viability of cells was assessed with Alamar blue assay. Each point on the graph is Mean ± SEM of 2 experiments done in sextuplicate. The IC_50_ was calculated by nonlinear regression analysis after converting the drug concentrations into log-X using Prism 5.0 software (GraphPad Software Inc.).

**Table 2: Characteristics of myeloma patients**

**Supplemental Figure 2: Sodium butyrate and doxorubicin combinations synergistically reduced the viability of myeloma cell lines. A)** 5x10^3^ NCI H929, RPMI 8226 or U266 cells were treated with increasing concentrations of butyrate (SB) (150, 300 and 600 μM) or doxorubicin (Dox) (15, 30 and 60 nM) or with their combinations. **B)** Alternatively cells were treated with a fixed concentration of doxorubicin (40 nM) and increasing concentrations of butyrate (0 - 800 μM) or with their combinations. Viability of cells was assessed with Alamar blue assay after 72 hr of treatment and the viability plots were derived by non-linear regression analysis after converting the drug concentrations to their log value (top panels). Each data point is an average of 2 independent experiments done in sextuplicate. The median effect of butyrate or doxorubicin combinations on each of these 3 cell lines was assessed by Chou and Talalay analysis with Calcusyn software (bottom panels).

**Supplemental Figure 3: Effect of pan-caspase inhibitor zVAD-FMK on butyrate and doxorubicin potentiated apoptosis.** 1x10^6^ RPMI 8226 cells were pretreated with 75 μM of ZVAD-FMK for 1 hr, followed by vehicle, butyrate (600 μM) doxorubicin (40 nM) or with their combination for 72 hr. Apoptotic index was determined by TUNEL assay.
